# Transcriptome Based Estrogen Related Genes Biomarkers for Diagnosis and Prognosis in Non-small Cell Lung Cancer

**DOI:** 10.3389/fgene.2021.666396

**Published:** 2021-04-14

**Authors:** Sinong Jia, Lei Li, Li Xie, Weituo Zhang, Tengteng Zhu, Biyun Qian

**Affiliations:** ^1^Hongqiao International Institute of Medicine, Shanghai Tongren Hospital and Faculty of Public Health, Shanghai Jiao Tong University School of Medicine, Shanghai, China; ^2^Clinical Research Institute, Shanghai Jiao Tong University School of Medicine, Shanghai, China; ^3^Shanghai Clinical Research Promotion and Development Center, Shanghai Hospital Development Center, Shanghai, China

**Keywords:** biomarker, mRNA, lung adenocarcinoma, lung squamous cell carcinoma, lung cancer

## Abstract

**Background:**

Lung cancer is the tumor with the highest morbidity and mortality, and has become a global public health problem. The incidence of lung cancer in men has declined in some countries and regions, while the incidence of lung cancer in women has been slowly increasing. Therefore, the aim is to explore whether estrogen-related genes are associated with the incidence and prognosis of lung cancer.

**Methods:**

We obtained all estrogen receptor genes and estrogen signaling pathway genes in The Cancer Genome Atlas (TCGA), and then compared the expression of each gene in tumor tissues and adjacent normal tissues for lung adenocarcinoma (LUAD) and lung squamous cell carcinoma (LUSC) separately. Survival analysis was performed of the differentially expressed genes in LUAD and LUSC patients separately. The diagnostic and prognostic values of the candidate genes were validated in the Gene Expression Omnibus (GEO) datasets.

**Results:**

We found 5 estrogen receptor genes and 66 estrogen pathway genes in TCGA. A total of 50 genes were differently expressed between tumor tissues and adjacent normal tissues and 6 of the 50 genes were related to the prognosis of LUAD in TCGA. 56 genes were differently expressed between tumor tissues and adjacent normal tissues and none of the 56 genes was related to the prognosis of LUSC in TCGA. GEO datasets validated that the 6 genes (*SHC1, FKBP4, NRAS, PRKCD, KRAS, ADCY9*) had different expression between tumor tissues and adjacent normal tissues in LUAD, and 3 genes (*FKBP4, KRAS, ADCY9*) were related to the prognosis of LUAD.

**Conclusions:**

The expressions of *FKBP4* and *ADCY9* are related to the pathogenesis and prognosis of LUAD. *FKBP4* and *ADCY9* may serve as biomarkers in LUAD screening and prognosis prediction in clinical settings.

## Introduction

Lung cancer is the tumor with the highest morbidity and mortality worldwide and china. There were about 2.1 million new cases and about 1.8 million lung cancer deaths all over the world in 2018 (Bray et al., 2018). This disease is a global public health problem. Studies have shown that in some countries and regions, the incidence of lung cancer in women has steadily increased, and the subtypes that women and men are susceptible to are different (Davis et al., 2013; Xie et al., 2020). The most common histological types are lung adenocarcinoma (LUAD) and lung squamous cell carcinoma (LUSC) in non-small cell lung cancer (NSCLC) (Skricková et al., 2018). Due to the heavy burden of lung cancer, biomarkers are of great value in the early diagnosis and treatment of lung cancer.

mRNA is the type of widely used biomarker in guiding clinical treatment and predicting the occurrence and prognosis of various cancers (Deng et al., 2014; Serilmez et al., 2019). For example, lung cancer patients with high *ERCC1* expression have significantly longer overall survival than those with low *ERCC1* expression (Simon et al., 2005). *BRCA1* is a prognostic biomarker in lung cancer, patients with high expression of *BRCA1* have a poor outcome (Karachaliou et al., 2013). In NSCLC, *PTEN* loses its function by downregulation via ubiquitin-mediated degradation (Li et al., 2015; Fan et al., 2020).

In recent years, the relationship between estrogen and tumor has attracted wide attention. Abnormal estrogen signal transduction can promote the occurrence of cancer and some metabolic diseases. Williams et al. (2016) found that the expression of Er β is significantly downregulated in colorectal cancer patients compared with normal tissues. Abnormal ER signaling pathways may change the biological function of the tumor by affecting the proliferation and invasion of the tumor. In breast cancer, the level of Er β was higher in normal breast tissue and decreased with the development of tumor from preinvasive tumor to tumor. In prostate cancer, studies have shown that estrogen antagonists inhibit the occurrence and development of prostate cancer in experimental and clinical conditions (Riggs and Hartmann, 2003). The roles of estrogen in the occurrence and development of lung cancer are widely discussed (Schwartz et al., 2005; Albain et al., 2007; Siegfried and Stabile, 2014). There are different opinions on the function and effect of estrogen in the occurrence and development of lung cancer (Ganti et al., 2006; Albain et al., 2007; Schwartz et al., 2007; Chlebowski et al., 2009; Navaratnam et al., 2012; Liu et al., 2013; Siegfried and Stabile, 2014). Studies have shown that serum estrogen level in patients with NSCLC is significantly higher than that in normal tissues, and serum estrogen level is related to tumor stage and prognosis. The higher the serum estrogen level, the later the tumor stage and the worse the prognosis (Eylem et al., 2018). The prognosis of NSCLC patients with ER – β expression is better, and the expression of Er – α is a risk factor for prognosis (Kawai et al., 2005). Although the expression of estrogen receptor (ER) is related to the histological type and differentiation degree of lung cancer (Mollerup et al., 2002), the relationship between the expression of estrogen receptor and the prognosis of lung cancer is controversial (Navaratnam et al., 2012; Liu et al., 2013; Lawrenson et al., 2015) and the diagnostic value of the estrogen receptor expression in lung cancer has not been widely studied. It is necessary to study the value of the expression of the genes that were involved in the estrogen signaling pathway and that encode estrogen receptors in the diagnosis and prognosis of lung cancer.

In this study, we compared the expression of the genes that encode estrogen receptors and that were involved in the estrogen signaling pathway between normal and tumor tissues in LUAD and LUSC based on The Cancer Genome Atlas (TCGA) database. We also evaluated the prognostic values of these genes for lung cancer in the TCGA database. The results were validated in the Gene Expression Omnibus (GEO) datasets.

## Materials and Methods

### Data Source

Lung cancer datasets in this study were obtained from TCGA (Tomczak et al., 2015) and GEO (Edgar et al., 2002). The lung cancer projects in TCGA contained lung adenocarcinoma (LUAD) and lung squamous cell carcinoma (LUSC). In the two projects, lung tumor tissues and lung normal tissues were extracted from the participants. The mRNA expression data of these samples were obtained by RNA sequencing. The clinical data of the participants contained the age of enrollment, sex, smoking status, pathological stage, TNM stage, and survival information. The TCGA data were used to explore the value of these biomarkers for the diagnosis and prognosis of lung cancer.

The dataset of GSE63459 (Robles et al., 2015) was obtained from GEO to validate the diagnosis value of the biomarkers for LUAD. There were lung tumor tissues and paired adjacent lung normal tissues of LUAD in this dataset. The mRNA expression data were generated by microarray. The age, sex, race, smoking status, the pathological stage was reported in this dataset.

The dataset of GSE68465 (Shedden et al., 2008) was used to validate the prognosis value of these biomarkers for LUAD. There were LUAD patients with the survival information in the dataset. The expression of mRNA was obtained through microarray. The clinical data contained histology, TNM stage, age, sex, race, smoking status, recurrence, and survival information.

### Estrogen Signaling Pathway Genes and Estrogen Receptor Genes

The genes in the estrogen signaling pathway were searched from the KEGG database (Kanehisa and Goto, 2000). We find the pathway of the “estrogen signaling pathway” that contained 66 genes (the KEGG id of this pathway was “map04915”). The estrogen receptor genes were searched from the GENE database of NCBI (Brown et al., 2014). There were 5 genes that encoded the estrogen receptors in the database. Supplementary Table 1 showed the gene list. In the TCGA mRNA datasets, 66 estrogen signaling pathway genes and 5 estrogen receptor genes were obtained for the analysis. Supplementary Table 1 summarized the information of these genes.

### Procedure of Screening the Genes That Were Differentially Expressed in Lung Cancer and Normal and That Were Related to the Prognosis of Lung Cancer

For the candidate genes, we first investigated whether they were differentially expressed between lung tumor tissues and normal lung tissues in TCGA datasets. This step was separately performed in the LUAD and LUSC datasets. We selected genes that have different expressions between normal and tumor tissues separately in LUAD further analysis. Next, we evaluated whether the expressions of the filtered genes were related to the survival of LUAD patients in TCGA datasets. We selected those genes that were differentially expressed in tumor tissues and normal tissues and that were related to the prognosis of LUAD patients as candidate genes for validation. Like the procedures performed in LUAD, the gene selection step was also performed in the LUSC dataset. Those genes, which were differentially expressed in tumor tissues and normal tissues and that were related to the prognosis of LUSC patients, were selected as candidate genes for validation.

### Validating the Diagnostic and Prognostic Value of the Candidate Genes for LUAD

Based on the GEO dataset, we validated the diagnostic and prognostic value of the genes filtered by previous steps. The GSE63459 dataset was used to validate the differential expression of the genes between LUAD tumor and adjacent normal tissues. We compared the expression of the candidate genes between lung tumors and adjacent normal tissues in LUAD. The GSE68465 dataset was used to validate the prognostic value of the genes in LUAD patients and the survival analyses were performed in this dataset. Because no gene passed the previous screening steps in the TCGA LUSC dataset, the validation steps were not performed in LUSC.

### Statistical Analyses

**FIGURE 1:**
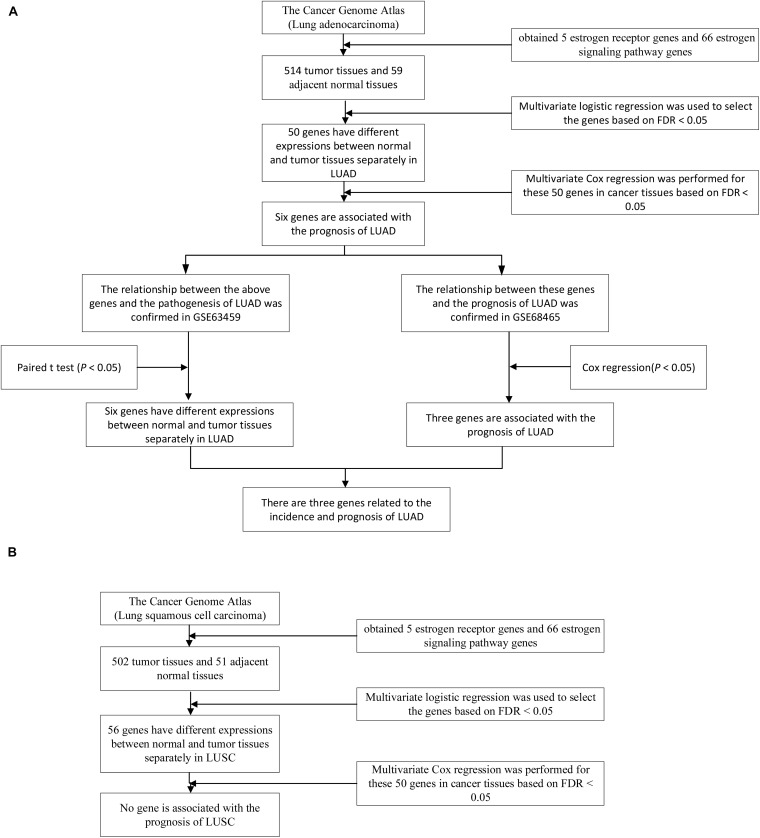
The whole research process of LUAD and LUSC. **(A)** A total of 50 genes were differentially expressed between tumor and normal tissues in TCGA LUAD dataset and 6 genes were associated with the survival of LUAD patients under the FDR < 0.05. The diagnostic values and the prognostic values of the 6 genes were validated the in the GSE63459 and GSE68465 respectively. **(B)** A total of 56 genes were differentially expressed between tumor and normal tissues in TCGA LUSC dataset. None of the 56 genes was associated with the survival of LUSC patients under the FDR < 0.05. Therefore, the validation steps were not performed for LUSC.

Subjects characters were described with means and 95% CIs (confidence intervals) for continuous variables and counts (percentages) for categorical variables. All expression data are normalized using zero-mean normalization. In TCGA, the different expression of the genes was tested using the multivariate logistic models by adjusting sex, age, pathological stage and smoking status (each logistic model contained one gene and other factors, including sex, age, pathological stage, and smoking status), and the logFC (log fold change) was calculated for each gene. Survival analysis was performed by the Cox models and sex, age, pathological stage, and smoking status were also adjusted in TCGA (each Cox model contained one gene’s expression value and other factors, including sex, age, pathological stage, and smoking status), and the HR (hazard ratio) was calculated for each gene. The false discovery rate (FDR) was used to counteract the problem of multiple comparisons. For each significant gene in survival analysis, we divided the patients into the high expression group and the low expression group by the expression of this gene, and the Kaplan-Meier survival curve was plotted for this gene. Since the tumor and normal samples in GSE63459 are paired, we used paired T-test to validate the differential expression of genes. Survival analysis was performed by the Cox models and sex, age, and smoking status were also adjusted in GSE68465 to validate the prognostic value (each Cox model contained one gene’s expression and other factors, including sex, age, and smoking status). The logFC and HR were also calculated for invalidation steps. For *ADCY9, FKBP4*, and *KRAS*, we divided the patients into the high expression group and the low expression group by the expression of each gene, and the Kaplan-Meier survival curves were plotted for these genes. All statistics were performed using R software (version 3.4.1)^1^. The whole procedure of the study was showed in Figure 1.

## Results

### Subjects Characters

There were 1016 lung cancer patients in TCGA datasets. Among them, 514 patients were LUAD and 502 patients were LUSC. 59 of the 514 LUAD patients had adjacent normal lung tissues and 51 LUSC patients had adjacent normal lung tissues. There were 276 females and 238 males in the LUAD patients with a mean age of 65. The LUAC dataset consisted of 131 females and 371 males with a mean age of 67. The detailed information about the subject characters of TCGA was showed in Table 1.

**TABLE 1:** Baseline of TCGA.

**Factor**	**LUAD**			**LUSC**		
	**Normal (*N* = 59)**	**Tumor (*N* = 514)**	***P***	**Normal (*N* = 51)**	**Tumor (*N* = 502)**	***P***
**Diagnosis age**	65.94 ± 10.91	65.82 ± 9.94	0.931	68.82 ± 8.60	67.72 ± 8.59	0.382
**Gender**			0.663			
Male	25 (42.37)	238 (46.30)		37 (72.55)	371 (73.90)	0.966
Female	34 (57.63)	276 (53.70)		14 (27.45)	131 (26.10)	
**Smoking history**			0.290			0.348
Never	7 (13.21)	74 (14.80)		0 (0.00)	18 (3.67)	
Current	8 (15.09)	119 (23.80)		13 (25.49)	133 (27.14)	
Ever	38 (71.70)	307 (61.40)		38 (74.51)	339 (69.18)	
**History of other malignancy**			0.339			0.889
No	45 (75.27)	423 (82.30)		45 (88.24)	434 (86.45)	
Yes	14 (23.73)	91 (17.70)		6 (11.76)	68 (13.56)	
**Tumor stage**			0.696			0.808
I	30 (51.72)	274 (54.15)		27 (52.94)	245 (49.20)	
II	13 (22.41)	122 (24.11)		17 (33.33)	162 (32.53)	
III	13 (22.41)	84 (16.60)		6 (11.76)	84 (16.87)	
IV	2 (3.45)	26 (5.14)		1 (1.96)	7 (1.41)	
**Pathologic T**			0.293			0.572
T1	19 (32.20)	169 (32.88)		9 (17.65)	114 (22.71)	
T2	37 (62.71)	276 (53.70)		35 (68.63)	294 (58.57)	
T3	2 (3.39)	47 (9.14)		5 (9.80)	71 (14.14)	
T4 & TX	1 (1.69)	22 (4.28)		2 (3.92)	23 (4.58)	
**Pathologic N**			0.078			0.953
N0	30 (50.85)	330 (64.33)		34 (66.67)	320 (63.75)	
N1	12 (20.34)	96 (18.71)		13 (25.49)	131 (26.10)	
N2	13 (22.03)	74 (14.42)		3 (5.88)	40 (8.00)	
N3 & NX	4 (6.78)	13 (2.53)		1 (1.96)	11 (2.19)	
**Pathologic M**			0.885			0.029
M0	40 (68.97)	345 (67.65)		33 (67.35)	412 (82.73)	
M1	2 (3.45)	25 (4.90)		1 (2.04)	7 (1.41)	
MX	16 (27.59)	140 (27.45)		15 (30.61)	79 (15.86)	

The GSE63459 had 31 lung tumor tissue and 31 paired adjacent lung normal tissues that were collected from 31 LUAD patients. The LUAD patients consisted of 16 females and 15 males and the mean age of them was 65. Most of the lung cancer patients were at the clinical stage one. We described the subject characters in Table 2.

**TABLE 2:** Baseline of dataset GSE63459 and GSE68465.

**Factor**	**GSE63459 (LUAD)**	**GSE68465 (LUAD)**
	
	**Tumor/adjacent normal (*N* = 31)**	**Tumor (*N* = 443)**
**Age**	65.90 ± 11.85	64.42 ± 10.10
**Gender**		
Male	15 (48.39)	223 (50.34)
Female	16 (51.61)	220 (49.66)
**Race**		
African	6 (19.35)	–
European	25 (80.65)	–
White	–	295 (67.51)
Other	–	142 (32.49)
**Smoking status**		
Never	4 (13.33)	49 (14.04)
Ever	26 (86.67)	268 (76.79)
Current	–	32 (9.17)
**Tumor stage**		
I	26 (86.67)	–
II	4 (13.33)	–
**Pathologic T**		
T1	–	150 (34.01)
T2	–	251 (56.92)
T3	–	28 (6.35)
T4	–	12 (2.72)
**Pathologic N**		
N0	–	299 (67.80)
N1	–	88 (19.95)
N2	–	53 (12.02)
NX	–	1 (0.23)
**Status**		
Alive	26 (83.87)	207 (46.73)
Dead	5 (16.13)	236 (53.27)
**Survival time^*a*^**	67.84 ± 40.49	171.91 ± 93.01
**Recurrence**		
No	–	157 (43.37)
Yes	–	205 (56.63)

*^*a*^Month.*

The GSE68465 had 443 LUAD patients that had the survival information. There were 220 females and 223 males among LUAD patients and the average age of them was 64. Over 50% of LUAD patients were stage one and stage two. The median survival time of LUAD patients was 14.2 years and 19 patients were censored. We described the subject characters in Table 2.

### The Genes That Were Differentially Expressed Between Lung Cancer and Normal in TCGA

Among the genes that were in the estrogen signaling pathway or that encoded estrogen receptor, 50 of them were differentially expressed between lung cancer tumor and normal tissues in the TCGA LUAD dataset. 49 differentially expressed genes were in the estrogen signaling pathway and the rest of them encoded estrogen receptor (Supplementary Table 2). In the LUSC dataset, 56 genes had different expressions between tumor tissues and normal tissues. 55 genes were in the estrogen signaling pathway and the rest of them encoded estrogen receptor (Supplementary Table 3).

### Genes That Were Related to the Prognosis of Lung Cancer

In the TCGA LUAD dataset, survival analysis of the 50 genes showed that 6 genes were associated with the survival of LUAD patients under the FDR < 0.05 (Supplementary Table 4).

**FIGURE 2:**
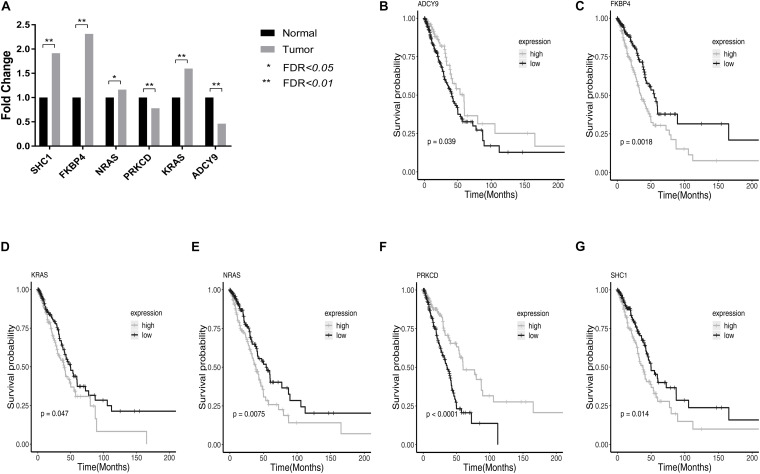
The relative expression and survival curves of the six genes with FDR < 0.05 in TCGA LUAD dataset. **(A)** The bar graph showed the relative expression of *PRKCD, ADCY9, SHC1, FKBP4, NRAS*, and *KRAS* among LUAD tumor tissues and normal tissues in TCGA LUAD dataset. **(B–G)** High expression of *PRKCD* and *ADCY9* prolonged the survival time of LUAD patients and the high expression of *SHC1, FKBP4, NRAS*, and *KRAS* reduced the survival time of LUAD patients.

Among them, *SHC1, FKBP4, NRAS, KRAS* had higher expression, and *PRKCD, ADCY9* had lower expression in LUAD tumor tissues compared with normal tissue. High expression of *PRKCD* and *ADCY9* can prolong the survival time of LUAD patients and the high expression of *SHC1, FKBP4, NRAS, and KRAS* can reduce the survival time of LUAD patients. The relative expression and survival curves of these 6 genes were plotted in Figure 2.

In the TCGA LUSC dataset, none of the 56 genes was associated with the survival of LUSC patients under the FDR < 0.05 (Supplementary Table 5).

### Validation Results of the Diagnostic and Prognostic Value of the 6 Genes for LUAD

**FIGURE 3:**
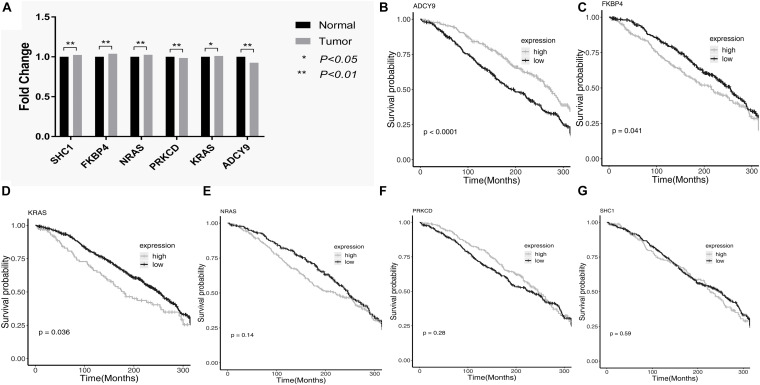
The relative expression and survival curves of these genes. **(A)** The bar graph showed the relative expression of *PRKCD, ADCY9, SHC1, FKBP4, NRAS* and *KRAS* among tumor tissues and normal tissues in the GSE63459. **(B–D)** The high expressions of *FKBP4* and *KRAS* reduced the survival time of LUAD patients and the high expressions of *ADCY9* prolonged the survival time of LUAD patients in the GSE68465. **(E–G)** In the GSE68465, *SHC1*, *NRAS* and *PRKCD* were not associated with the prognosis of LUAD.

The diagnostic values of the 6 genes were validated in the GSE63459. The *SHC1, FKBP4, NRAS, and KRAS* had higher expressions in lung cancer tissues compared with lung normal tissues in LUAD. The *PRKCD* and *ADCY9* had lower expression in lung normal tissues compared with lung cancer tissues in LUAD. Figure 3 showed the relative expression of these genes in LUAD.

The prognostic values of the 6 genes were validated in the GSE68465 (Supplementary Table 6). The validation results showed that the *FKBP4* and *KRAS* were the risk factors for the survival of LUAD patients and the high expressions of *FKBP4* and *KRAS* reduced the survival time of LUAD patients. The *ADCY9* was the protective factor for the survival of LUAD patients and the high expressions of *ADCY9* prolonged the survival time of LUAD patients. In this dataset, *SHC1, NRAS* and *PRKCD* were not associated with the prognosis of LUAD. Figure 3 showed the survival curves of these genes in LUAD.

No genes passed the screening steps in the TCGA LUSC dataset. Therefore, the validation steps were not performed for LUSC.

## Discussion

In this study, we focused on the mRNA expression of the genes that encode estrogen receptors and the genes in the estrogen signaling pathway. A total of 71 genes were found from the KEGG database and gene database of NCBI. Among these genes, 50 genes were differently expressed between lung cancer tissues and lung normal tissues in LUAD, and 56 genes were differently expressed in LUSC in TCGA. Among the 50 differentially expressed genes in LUAD, *FKBP4*, *ADCY9*, and *KRAS* were associated with the prognosis of LUAD. No gene was found to be associated with the prognosis of LUSC in TCGA. The different expressions and the prognostic value of *FKBP4*, *ADCY9*, and *KRAS* in LUAD were validated in the GEO datasets. Our study suggested that high-expression of *FKBP4* and low-expression of *ADCY9* were the risks of LUAD and reduced the overall survival time of LUAD patients. Overexpression of *KRAS* was a risk of LUAD and reduced the overall survival time of LUAD patients.

The functions of *KRAS* in LUAD have been widely discussed (Ferrer et al., 2018) and the results of *KRAS* in our study were completely consistent with previous reports (Birkeland et al., 2012; Liang et al., 2012; Guin et al., 2013; Yang and Kim, 2018). *KRAS* played as a positive marker to confirm the credibility of our research method.

The *FKBP4* (*FKBP4* is also known as *FKBP52*) is located in 12p13.33, gene expression is influenced by both genetic and epigenetic mechanisms (Cioffi et al., 2011). The expression of this gene is the highest in the testis tissue with a mean RPKM (Reads Per Kilobase per Million mapped reads) of 24.92 and the mean RPKM of the *FKBP4* in lung tissue is 7.71 (Fagerberg et al., 2014). The protein of *FKBP4* is a member of the immunophilin protein family (Peattie et al., 1992), which play a role in immunoregulation and basic cellular processes involving protein folding and trafficking (Zgajnar et al., 2019). The FKBP4-Hsp90 complex regulates the nuclear-initiated steroid signaling of the estrogen receptors in the estrogen signaling pathway (Solassol et al., 2011; Mangé et al., 2019). *FKBP4* acts as a specific positive regulator of androgen receptors, glucocorticoid receptors, and progesterone receptors function through the interaction of the proline-rich loop with the ligand-binding domain of the steroid hormone receptors (Guy et al., 2015). *FKBP4* showed significantly increased reactivity in primary breast cancer and carcinoma *in situ* compared with healthy controls (Desmetz et al., 2009) and might be putative prediction markers in discriminating malignant (Xiong et al., 2020) and drug-resistant of breast cancers (Ostrow et al., 2009; Yang W.S. et al., 2012). *FKBP4* was found to be overexpressed in prostate cancer (Lin et al., 2007) and hepatocellular carcinoma (Liu et al., 2010) compared to control. *FKBP4* was significant with a high fold change in oral cancer (Mohanta et al., 2019). The expression of *FKBP4* was up-regulated in epithelial ovarian cancer and higher *FKBP4* expression was associated with significantly worse overall survival (Lawrenson et al., 2015). Compared with controls, *FKBP4* mRNA expression was decreased in the endometrium of women with endometriosis (Yang H. et al., 2012). The rs12582595 of *FKBP4* was correlated with general health improvement in systemic lupus erythematosus patients (Lou et al., 2020). The *FKBP4* SNP rs4409904 was associated with lower odds of polycystic ovary syndrome. *FKBP4* is likely to have an important role and to serve as a therapeutic target in a variety of diseases that are dependent on these hormone signaling pathways (Storer et al., 2011). In general, the expression of *FKBP4* is increased in tumor tissues, and overexpression *FKBP4* indicates a poor prognosis for cancer patients. In other non-cancer diseases, the expression of *FKBP4* shows different trends in different diseases. In our study, the expression of *FKBP4* was up-regulated in LUAD, and patients with high *FKBP4* expression had a relatively poor prognosis, which was the first report in LUAD and was consistent with the expression changes in other cancers.

The *ADCY9* (which is also known as *AC9* or *ACIX*) is located in 16p13.3, which is regulated by a family of G protein-coupled receptors (Choi et al., 2010), protein kinases, and calcium (Cumbay and Watts, 2004). The *ADCY9* has the highest expression in the thyroid (RPKM 11.2) and it also has a relatively high expression in lung tissue with an RPKM of 8.5 (Fagerberg et al., 2014). Some studies focused on the association between *ADCY9* and the risk of cardiovascular disease. Wu Y. et al. reported that deletion of *ADCY9* is the causation for the cardiac abnormalities (Li et al., 2020). SNP rs2238432 in the *ADCY9* gene was linked with decreased stroke risk (Flanagan et al., 2011). Proportional reductions in the risk of major vascular events with anacetrapib did not differ significantly by *ADCY9* rs1967309 genotype (Hopewell et al., 2019). There was no significant association between the *ADCY9* rs1967309 genotype and cardiovascular benefit or harm for the cholesteryl ester transfer protein inhibitor evacetrapib (Nissen et al., 2018). The expression of *ADCY9* was downregulated in intracranial aneurysms (Lai and Du, 2019; Li et al., 2020). In comparison to the healthy controls, granulomatosis with polyangiitis patients had lower *ADCY9* mRNA levels (Dekkema et al., 2019). Some studies found that *ADCY9* is associated with respiratory diseases. Respiratory distress syndrome was associated with fetal single nucleotide polymorphisms in *ADCY9* (Haas et al., 2012). Some studies evaluated the different responses to treatment in different *ADCY9* Genotypes. Kim et al. (2011) suggested that *ADCY9* gene polymorphisms may alone, and in combination with *ADRB2* gene polymorphisms, contribute to individual response to combination therapy in mild to moderate asthmatics. Some studies have focused on the relationship between *ADCY9* and the incidence and prognosis of cancer. One study discovered the association of *ADCY9* variants with glioma risk and prognosis (Zhang et al., 2020) and the other two studies suggested *ADCY9* gene polymorphisms were associated with Hepatocellular Carcinoma (Chao et al., 2020) and colorectal cancer (Li et al., 2020) risk in the Chinese Han population. Several studies reported the relationship between *ADCY9* expression and disease risk and prognosis. *ADCY9* had hypermethylation and low-expression in bladder cancer (Zhang et al., 2018). Orchel, J. *et al.* suggested that *ADCY9* had lower expression in grade 1 and grade 2 patients and higher expression in grade 3 patients than in the controls in endometrial cancer (Orchel et al., 2012). *ADCY9* had a different expression between *EGFR/KRAS* mutation groups in LUAD (Planck et al., 2013). One study showed that *ADCY9* immunoreactivity scores were significantly higher (*P* = 0.002) in tumor tissues than in adjacent normal samples in colon cancer, and *ADCY9* high expression level was associated with poor disease-free survival (*P* = 0.001) but not overall survival (*P* = 0.055) (Yi et al., 2018) in colon cancer. The results of the study about colon cancer (Yi et al., 2018) were contrary to our results. It is common for a gene to play different roles in different cancers. However, the study about colon cancer (Yi et al., 2018) had some limitations; for instance, there were 200 cancer samples and only 8 adjacent normal colon tissues in immunoreactivity scores analysis, which meant the sample size was unbalanced (Yi et al., 2018). In our study, the expression of *ADCY9* was down-regulated in LUAD, and patients with high *ADCY9* expression had a relatively better prognosis. The findings of our study were the first to be reported in LUAD and were consistent with the expression changes in most other cancers.

The direct relationship between estrogen and *FKBP4* in lung cancer has not been fully elucidated. However, under the influence of estrogen, the mRNA and protein of *FKBP4* were up-regulated in breast cancer cells (Kumar et al., 2001; Collodoro et al., 2012). This study demonstrated the mRNA of *FKBP4* was up-regulated in LUAD. The FKBP4-Hsp90 protein complex regulated the nuclear-initiated steroid signaling of the estrogen receptors (Solassol et al., 2011; Mangé et al., 2019). The up-regulated FKBP4-Hsp90 protein complex initiated the signals of nuclear-initiated steroid signaling action of estrogen receptors, leading to the activation of AKT and mitogen-activated protein kinase (AKT/MAPK) signaling pathways (Song, 2007). The activation of AKT/MAPK signaling pathways may promote the initiation and development of lung cancer and may make a poor prognosis for lung cancer patients. The ADCY9 protein is a membrane-bound enzyme that catalyzes the formation of cyclic Adenosine monophosphate (cAMP) from Adenosine triphosphate (ATP) (Hacker et al., 1998) in the estrogen signaling pathway. *ADCY9* protein is affected by changes in membrane-rich cholesterol plasma membrane domains (Niesor and Benghozi, 2015). Reduced amplitude of cAMP circadian oscillation was probably associated with changed expression of *ADCY9* (Baburski et al., 2017). *ITGA1* and *ADCY9* competed for binding to *miR-181b*, and *ZEB1* upregulated *ITGA1* to activate a miR-181b–regulated ceRNA network that increased metastasis through *ADCY9* (Tan et al., 2018) in LUAD. The expression of *ADCY9* was regulated by estradiol in the human MCF-7 breast cancer cell line (Deroo et al., 2009). *ADCY9* had high methylation in chronic alcohol consumption people (Weng et al., 2015), which resulted in down-regulation of *ADCY9* expression. Under the stimulation of the internal and external environment, the *ADCY9* expression may change with the occurrence and development of lung cancer.

In normal lung tissue, estrogen receptor beta (ERβ, also known as ESR2) is highly expressed in pneumocytes and the bronchial epithelial cells (Brandenberger et al., 1997). Estrogen receptors (ER) are consistently found in lung cancer tissues and cell lines, especially adenocarcinoma, and mostly in the form of the *ER*β (Hsu et al., 2017). ER-alpha (ERα, also known as ESR1) mRNA and protein are expressed at extremely low levels in the lung tissues (Stabile and Siegfried, 2004). Fasco et al. reported that *ER*α expression occurred more often in the lungs of women than men, whereas *ER*β was expressed with approximately equal frequency in the lungs of both genders, and lung tumors displayed a higher expression frequency of both receptor types than non-tumorous in women (Fasco et al., 2002). Zhang et al. suggested that knockdown of *ER*β by short hairpin RNA constructs resulted in the loss of estrogen-dependent growth of lung cancer cells (Zhang et al., 2009). However, Kawai et al. reported *ER*α expression and the absence of *ER*β expression were associated with a poorer prognosis among NSCLC patients. There were conflicting results about the effect of estrogen expression on the risk and/or survival of lung cancer (Hsu et al., 2017). Our study included the mRNA expressions of *ER*α, which are highly expressed in both LUAD and LUSC tissue with the *P* values was 0.08 and 0.01 respectively, and the mRNA expressions of *ER*β, which are highly expressed in both LUAD and LUSC tissue with the *p* values of 0.04 and 0.07 respectively. However, they were not selected as candidates according to our analysis process. Those genes with differential expression in lung cancer and normal tissues and meet the FDR < 0.05 can be further analyzed. The *ER*α and *ER*β did not meet the criteria.

In this study, *FKBP4*, *ADCY9*, and *KRAS* were differentially expressed in cancer tissues and adjacent normal tissues of LUAD and were related to the prognosis of LUAD. However, no gene was not only differentially expressed in cancer tissues and adjacent normal tissues but also related to prognosis in LUSC. There are many reasons for this result. First of all, the number of normal tissues in the TCGA database is far less than that of lung cancer. This may affect the effectiveness of statistical tests. Secondly, The different sex ratio between the two subtypes of lung cancer (Radkiewicz et al., 2019) points out a possibility that sex-related factors may have different effects on the two subtypes of lung cancer. Although both occur in lung tissues, these two subtypes show several different pathological characteristics (Liu et al., 2018). Estrogen receptor α and β are prognostic factors in NSCLC (Kawai et al., 2005). The adverse effects of estrogen on the prognosis of LUAD have been discussed (Hammoud et al., 2008; Weng et al., 2015). Hormone replacement therapy (HRT) has been examined about lung cancer incidence and mortality (Siegfried et al., 2009; Rodriguez-Lara et al., 2018). However, there are few reports about the relationship between estrogen-related factors (including estrogen, estrogen receptor, and estrogen signaling pathway) and LUSC. The increase of CYFRA21-1 is a risk factor for the prognosis of recurrent and metastatic LUSC (Zhang et al., 2017). *POK3CA* mutation may be related to the prognosis of lung squamous cell carcinoma (Paik et al., 2015). *NRF2* mutation is a risk factor for the prognosis of lung squamous cell carcinoma (Sasaki et al., 2013). Lung squamous cell carcinoma and adenocarcinoma are different not only in genetic and gene-phenotype but also in biological behavior. The molecular mechanisms of LUAD and LUSC could be highly different (Cancer Genome Atlas Research Network, 2012, 2014). There may be no direct relationship between estrogen and LUSC. Therefore, there were no genes related to the incidence and mortality of LUSC in our study. Since LUAD and LUSC have significantly different clinical characters and outcomes in lung cancer, researchers suggest these two different cancers should be analyzed separately to provide more precise outcomes (Wang et al., 2020).

This study had some limitations. First, the relationships between gene expression and lung cancer were only correlations, and whether there were causal relationships between them had not been explored. Second, the number of normal tissue in the TCGA database is far less than that of lung cancer tissue. This may affect the power of statistical tests. Third, the underlying biological mechanisms of this gene in increasing the risk of lung cancer and affecting the survival time of lung cancer patients had not been explored. Last, we did not carry out vitro experiments to confirm the relationship between the genes and LUAD. In the future, we will conduct more research to explore the role of *ADCY9* and *FKBP4* in LUAD.

## Conclusion

The *FKBP4* is a risk factor of LUAD and the high expression of *FKBP4* reduces the survival time of LUAD patients. The *ADCY9* is a protective factor of LUAD and high expression of *ADCY9* prolongs the survival time of LUAD patients. *FKBP4* and *ADCY9* may serve as biomarkers and have potential values in the diagnosis and prognosis of LUAD.

## Data Availability Statement

Publicly available datasets were analyzed in this study. This data can be found here: https://www.cancer.gov/about-nci/organization/ccg/research/structural-genomics/tcga; https://www.ncbi.nlm.nih.gov/geo/query/acc.cgi?acc=GSE63459; and https://www.ncbi.nlm.nih.gov/geo/query/acc.cgi?acc=GSE68465.

## Author Contributions

SJ, LL, and BQ designed the study. LX and BQ coordinated the study. SJ and LL performed the acquisition of data and the statistical analysis, and drafted the manuscript. SJ, LX, WZ, and BQ interpreted the data. All authors revised the final manuscript and approved this version to be published.

## Conflict of Interest

The authors declare that the research was conducted in the absence of any commercial or financial relationships that could be construed as a potential conflict of interest. The handling editor declared a shared affiliation, though no other collaboration, with several of the authors, SJ, LL, LX, WZ, and TZ.

## Footnotes

1 https://cran.r-project.org/bin/windows/base/old/3.4.1/
