# Supplementary figures and images for: Transcriptome Based Estrogen Related Genes Biomarkers for Diagnosis and Prognosis in Non-small Cell Lung Cancer
Source: Front Genet. 2021 Apr 14;12:666396. doi: 10.3389/fgene.2021.666396 (PMC8081391; doi:10.3389/fgene.2021.666396)

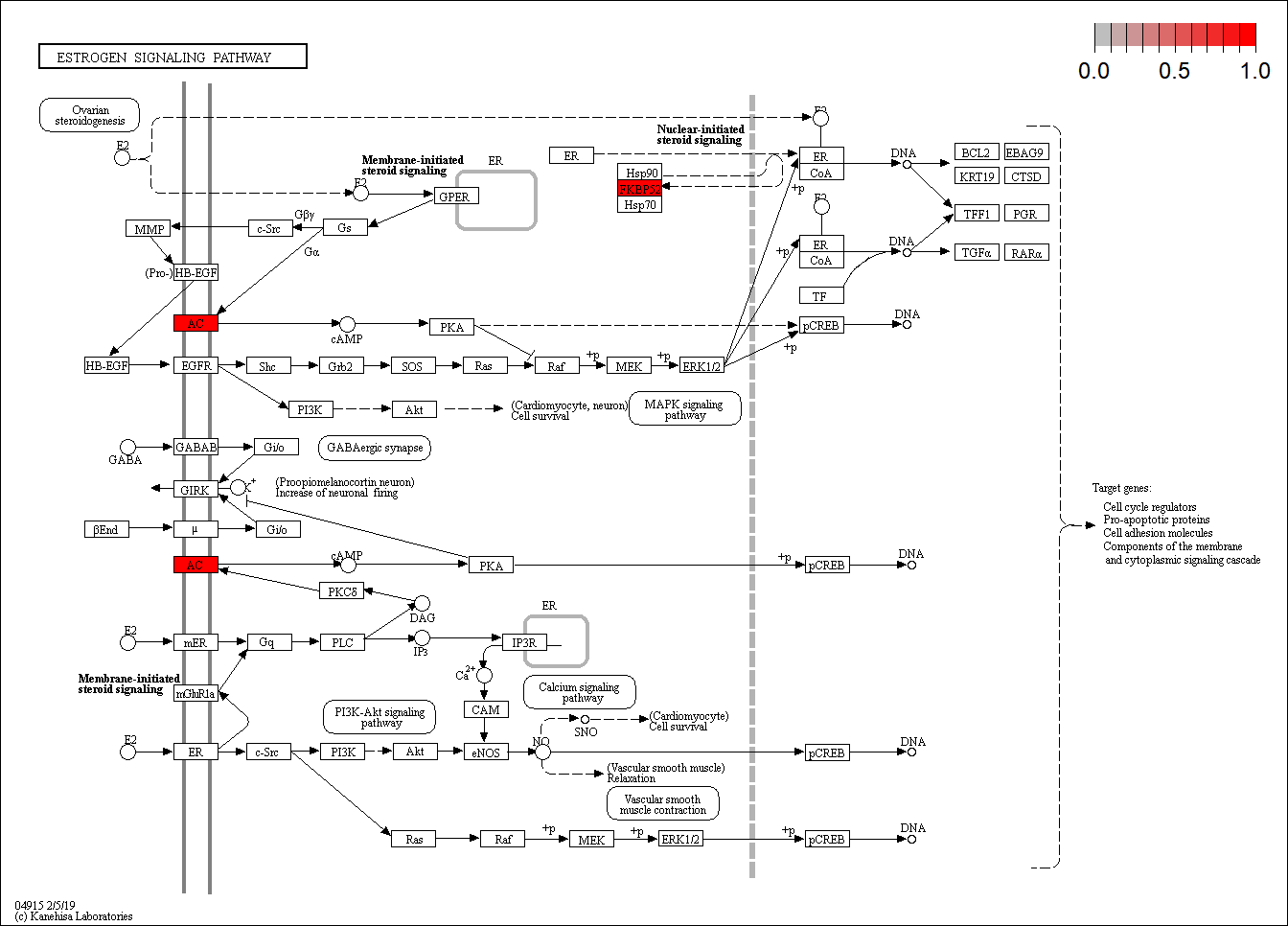

Supplement: Supplementary file 1 [file Image_1.PNG]
