# Supplementary material for: Transcriptome Based Estrogen Related Genes Biomarkers for Diagnosis and Prognosis in Non-small Cell Lung Cancer
Source: Front Genet. 2021 Apr 14;12:666396. doi: 10.3389/fgene.2021.666396 (PMC8081391; doi:10.3389/fgene.2021.666396)
Supplement: Supplementary file 2 [file Table_1.docx]

Table S1. Gene names of Estrogen Pathway and Receptor

| **Gene** | **Full name** | **Type** |
| --- | --- | --- |
| *ADCY1* | adenylate cyclase 1 | Pathway |
| *ADCY2* | adenylate cyclase 2 | Pathway |
| *ADCY3* | adenylate cyclase 3 | Pathway |
| *ADCY4* | adenylate cyclase 4 | Pathway |
| *ADCY5* | adenylate cyclase 5 | Pathway |
| *ADCY6* | adenylate cyclase 6 | Pathway |
| *ADCY7* | adenylate cyclase 7 | Pathway |
| *ADCY8* | adenylate cyclase 8 | Pathway |
| *ADCY9* | adenylate cyclase 9 | Pathway |
| *AKT2* | AKT serine/threonine kinase 2 | Pathway |
| *ATF6B* | cyclic AMP-dependent transcription factor ATF-6 beta | Pathway |
| *BCL2* | apoptosis regulator Bcl-2 | Pathway |
| *CREB1* | cyclic AMP-responsive element-binding protein 1 | Pathway |
| *CREB3* | cyclic AMP-responsive element-binding protein 3 | Pathway |
| *CREB5* | cyclic AMP-responsive element-binding protein 5 | Pathway |
| *CTSD* | cathepsin D | Pathway |
| *EGFR* | epidermal growth factor receptor | Pathway |
| *FKBP4* | FK506-binding protein 4 | Pathway |
| *FKBP5* | FK506-binding protein 5 | Pathway |
| *FOS* | proto-oncogene protein c-fos | Pathway |
| *GNAS* | guanine nucleotide-binding protein G(s) subunit alpha | Pathway |
| *GRB2* | growth factor receptor-bound protein 2 | Pathway |
| *GRM1* | metabotropic glutamate receptor 1 | Pathway |
| *HBEGF* | heparin-binding EGF-like growth factor | Pathway |
| *HRAS* | GTPase HRas | Pathway |
| *ITPR1* | inositol 1,4,5-triphosphate receptor type 1 | Pathway |
| *ITPR2* | inositol 1,4,5-triphosphate receptor type 2 | Pathway |
| *ITPR3* | inositol 1,4,5-triphosphate receptor type 3 | Pathway |
| *JUN* | transcription factor AP-1 | Pathway |
| *KCNJ3* | potassium inwardly-rectifying channel subfamily J member 3 | Pathway |
| *KCNJ5* | potassium inwardly-rectifying channel subfamily J member 5 | Pathway |
| *KCNJ6* | potassium inwardly-rectifying channel subfamily J member 6 | Pathway |
| *KCNJ9* | potassium inwardly-rectifying channel subfamily J member 9 | Pathway |
| *KRAS* | GTPase KRas | Pathway |
| *KRT1* | type I keratin, acidic | Pathway |
| *MAP2K1* | mitogen-activated protein kinase kinase 1 | Pathway |
| *MAP2K2* | mitogen-activated protein kinase kinase 2 | Pathway |
| *MAPK1* | mitogen-activated protein kinase 1 | Pathway |
| *MAPK3* | mitogen-activated protein kinase 3 | Pathway |
| *MMP2* | matrix metalloproteinase-2 (gelatinase A) | Pathway |
| *MMP9* | matrix metalloproteinase-9 (gelatinase B) | Pathway |
| *NCOA1* | nuclear receptor coactivator 1 | Pathway |
| *NCOA2* | nuclear receptor coactivator 2 | Pathway |
| *NCOA3* | nuclear receptor coactivator 3 | Pathway |
| *NOS3* | nitric-oxide synthase, endothelial | Pathway |
| *NRAS* | GTPase NRas | Pathway |
| *OPRM1* | mu-type opioid receptor | Pathway |
| *PGR* | progesterone receptor | Pathway |
| *PIK3CA* | phosphatidylinositol-4,5-bisphosphate 3-kinase catalytic subunit alpha | Pathway |
| *PIK3CB* | phosphatidylinositol-4,5-bisphosphate 3-kinase catalytic subunit beta | Pathway |
| *PIK3CD* | phosphatidylinositol-4,5-bisphosphate 3-kinase catalytic subunit delta | Pathway |
| *PIK3R1* | phosphoinositide-3-kinase regulatory subunit alpha | Pathway |
| *PIK3R2* | phosphoinositide-3-kinase regulatory subunit beta | Pathway |
| *PIK3R3* | phosphoinositide-3-kinase regulatory subunit delta | Pathway |
| *POMC* | proopiomelanocortin | Pathway |
| *PRKCD* | novel protein kinase C delta type | Pathway |
| *RAF1* | RAF proto-oncogene serine/threonine-protein kinase | Pathway |
| *RARA* | retinoic acid receptor alpha | Pathway |
| *SHC1* | SHC-transforming protein 1 | Pathway |
| *SHC2* | SHC-transforming protein 2 | Pathway |
| *SHC3* | SHC-transforming protein 3 | Pathway |
| *SHC4* | SHC-transforming protein 4 | Pathway |
| *SP1* | transcription factor Sp1 | Pathway |
| *SRC* | tyrosine-protein kinase Src | Pathway |
| *TFF1* | trefoil factor 1 | Pathway |
| *TGFA* | transforming growth factor, alpha | Pathway |
| *ESR1* | estrogen receptor alpha | Receptor |
| *ESR2* | estrogen receptor beta | Receptor |
| *ESRRA* | estrogen related receptor alpha | Receptor |
| *ESRRG* | estrogen related receptor gamma | Receptor |
| *ESRRB* | estrogen related receptor beta | Receptor |
