# Supplementary material for: Transcriptome Based Estrogen Related Genes Biomarkers for Diagnosis and Prognosis in Non-small Cell Lung Cancer
Source: Front Genet. 2021 Apr 14;12:666396. doi: 10.3389/fgene.2021.666396 (PMC8081391; doi:10.3389/fgene.2021.666396)
Supplement: Supplementary file 3 [file Table_2.docx]

Table S2. Differential expression genes of lung adenocarcinoma in TCGA database

| **Gene** | **logFC** | ***P* value** | **FDR** | **seq** |
| --- | --- | --- | --- | --- |
| *ADCY4* | -1.88743 | 1.28E-16 | 3.83E-15 | 1 |
| *PIK3R1* | -1.08526 | 4.52E-14 | 6.79E-13 | 2 |
| *HBEGF* | -2.52778 | 1.37E-13 | 1.37E-12 | 3 |
| *PGR* | -1.7953 | 3.15E-12 | 2.36E-11 | 4 |
| *ADCY9* | -1.1185 | 3.50E-12 | 2.10E-11 | 5 |
| *SHC1* | 0.936147 | 1.50E-11 | 7.52E-11 | 6 |
| *FOS* | -1.64081 | 1.67E-11 | 7.17E-11 | 7 |
| *JUN* | -1.01587 | 4.81E-11 | 1.81E-10 | 8 |
| *ADCY8* | -3.33638 | 9.95E-10 | 3.32E-09 | 9 |
| *KCNJ5* | -1.69155 | 1.90E-09 | 5.69E-09 | 10 |
| *FKBP4* | 1.209193 | 2.63E-09 | 7.19E-09 | 11 |
| *PIK3R2* | 0.604586 | 2.93E-09 | 7.34E-09 | 12 |
| *RARA* | -0.55083 | 1.12E-08 | 2.59E-08 | 13 |
| *ATF6B* | 0.489203 | 3.71E-08 | 7.96E-08 | 14 |
| *SRC* | 0.612843 | 1.26E-07 | 2.52E-07 | 15 |
| *MAPK3* | -0.36856 | 8.73E-07 | 1.64E-06 | 16 |
| *KRAS* | 0.675106 | 2.36E-06 | 4.17E-06 | 17 |
| *SHC3* | -0.96362 | 4.57E-06 | 7.62E-06 | 18 |
| *TGFA* | 1.490159 | 5.55E-06 | 8.76E-06 | 19 |
| *NCOA1* | -0.54125 | 6.64E-06 | 9.96E-06 | 20 |
| *MMP9* | 2.14045 | 6.82E-06 | 9.75E-06 | 21 |
| *PIK3R3* | -0.75574 | 1.22E-05 | 1.66E-05 | 22 |
| *ITPR1* | -0.72331 | 2.52E-05 | 3.28E-05 | 23 |
| *ESRRA* | 0.470815 | 3.30E-05 | 4.12E-05 | 24 |
| *GNAS* | 0.482043 | 4.60E-05 | 5.52E-05 | 25 |
| *KRT1* | -1.74115 | 1.32E-04 | 1.52E-04 | 26 |
| *PRKCD* | -0.35631 | 1.90E-04 | 2.11E-04 | 27 |
| *GRB2* | -0.17816 | 6.74E-04 | 7.22E-04 | 28 |
| *AKT2* | 0.25686 | 6.77E-04 | 7.01E-04 | 29 |
| *CTSD* | -0.35144 | 7.31E-04 | 7.31E-04 | 30 |
| *TFF1* | 9.626475 | 1.28E-03 | 1.24E-03 | 31 |
| *MAP2K2* | 0.235381 | 3.78E-03 | 3.55E-03 | 32 |
| *CREB3* | 0.291463 | 4.76E-03 | 4.33E-03 | 33 |
| *CREB1* | -0.16041 | 7.66E-03 | 6.76E-03 | 34 |
| *NCOA3* | 0.205884 | 9.29E-03 | 7.96E-03 | 35 |
| *ESR2* | 0.373396 | 1.23E-02 | 1.03E-02 | 36 |
| *ITPR3* | 0.361996 | 1.24E-02 | 1.00E-02 | 37 |
| *CREB5* | -0.60485 | 1.36E-02 | 1.07E-02 | 38 |
| *MAPK1* | -0.2068 | 2.62E-02 | 2.01E-02 | 39 |
| *KCNJ6* | 2.735568 | 2.82E-02 | 2.12E-02 | 40 |
| *FKBP5* | -0.38297 | 3.43E-02 | 2.51E-02 | 41 |
| *ADCY7* | -0.31349 | 3.61E-02 | 2.58E-02 | 42 |
| *PIK3CB* | 0.160762 | 3.94E-02 | 2.75E-02 | 43 |
| *OPRM1* | 3.265379 | 4.57E-02 | 3.12E-02 | 44 |
| *NCOA2* | 0.238528 | 4.61E-02 | 3.07E-02 | 45 |
| *PIK3CA* | -0.20974 | 4.68E-02 | 3.05E-02 | 46 |
| *SHC2* | 0.441336 | 4.89E-02 | 3.12E-02 | 47 |
| *HRAS* | 0.252935 | 5.15E-02 | 3.22E-02 | 48 |
| *ESR1* | 0.993294 | 7.71E-02 | 4.72E-02 | 49 |
| *NRAS* | 0.218817 | 7.97E-02 | 4.78E-02 | 50 |
