# Supplementary material for: Transcriptome Based Estrogen Related Genes Biomarkers for Diagnosis and Prognosis in Non-small Cell Lung Cancer
Source: Front Genet. 2021 Apr 14;12:666396. doi: 10.3389/fgene.2021.666396 (PMC8081391; doi:10.3389/fgene.2021.666396)
Supplement: Supplementary file 4 [file Table_3.docx]

Table S3. Differential expression genes of lung squamous cell carcinoma in TCGA database

| **Gene** | **logFC** | ***P* value** | **FDR** | **seq** |
| --- | --- | --- | --- | --- |
| *RARA* | -1.55E+00 | 3.53E-19 | 2.51E-17 | 1 |
| *PRKCD* | -9.96E-01 | 9.66E-19 | 3.43E-17 | 2 |
| *ADCY9* | -1.68E+00 | 2.54E-18 | 6.01E-17 | 3 |
| *SHC3* | -2.12E+00 | 2.77E-18 | 4.92E-17 | 4 |
| *ITPR1* | -1.75E+00 | 5.90E-18 | 8.38E-17 | 5 |
| *PGR* | -2.82E+00 | 6.75E-18 | 7.98E-17 | 6 |
| *ADCY4* | -2.73E+00 | 4.75E-17 | 4.82E-16 | 7 |
| *MAPK3* | -8.26E-01 | 1.16E-16 | 1.03E-15 | 8 |
| *ADCY6* | -1.00E+00 | 2.60E-16 | 2.05E-15 | 9 |
| *KCNJ5* | -1.95E+00 | 6.86E-16 | 4.87E-15 | 10 |
| *CTSD* | -1.17E+00 | 1.91E-15 | 1.23E-14 | 11 |
| *HBEGF* | -1.70E+00 | 2.02E-15 | 1.19E-14 | 12 |
| *ADCY7* | -8.65E-01 | 2.74E-15 | 1.50E-14 | 13 |
| *FOS* | -2.44E+00 | 5.50E-15 | 2.79E-14 | 14 |
| *PIK3R2* | 1.21E+00 | 5.47E-13 | 2.59E-12 | 15 |
| *HRAS* | 1.29E+00 | 7.09E-13 | 3.14E-12 | 16 |
| *JUN* | -1.18E+00 | 9.85E-13 | 4.11E-12 | 17 |
| *SRC* | 8.04E-01 | 1.41E-11 | 5.56E-11 | 18 |
| *TGFA* | 1.85E+00 | 4.57E-10 | 1.71E-09 | 19 |
| *ITPR2* | -9.10E-01 | 3.13E-09 | 1.11E-08 | 20 |
| *PIK3CA* | 9.37E-01 | 4.73E-09 | 1.60E-08 | 21 |
| *AKT2* | 7.12E-01 | 4.76E-09 | 1.53E-08 | 22 |
| *KRAS* | 7.10E-01 | 1.21E-08 | 3.73E-08 | 23 |
| *SHC1* | 7.94E-01 | 1.54E-07 | 4.56E-07 | 24 |
| *MAP2K2* | 4.06E-01 | 2.82E-07 | 8.01E-07 | 25 |
| *MMP9* | 2.42E+00 | 4.50E-07 | 1.23E-06 | 26 |
| *FKBP4* | 2.20E+00 | 6.04E-07 | 1.59E-06 | 27 |
| *RAF1* | -2.64E-01 | 8.93E-07 | 2.26E-06 | 28 |
| *FKBP5* | -1.10E+00 | 9.69E-07 | 2.37E-06 | 29 |
| *ESRRA* | 5.98E-01 | 1.12E-06 | 2.65E-06 | 30 |
| *ADCY3* | 6.08E-01 | 1.20E-06 | 2.74E-06 | 31 |
| *EGFR* | 1.63E+00 | 1.34E-06 | 2.98E-06 | 32 |
| *NRAS* | 5.09E-01 | 1.44E-06 | 3.10E-06 | 33 |
| *GNAS* | 4.36E-01 | 2.40E-06 | 5.01E-06 | 34 |
| *NCOA1* | -4.03E-01 | 3.26E-06 | 6.62E-06 | 35 |
| *PIK3R3* | -7.49E-01 | 1.25E-05 | 2.46E-05 | 36 |
| *PIK3CB* | 4.90E-01 | 1.45E-05 | 2.78E-05 | 37 |
| *PIK3R1* | -7.51E-01 | 1.71E-04 | 3.19E-04 | 38 |
| *NCOA3* | 4.41E-01 | 2.78E-04 | 5.07E-04 | 39 |
| *GRB2* | -1.97E-01 | 2.89E-04 | 5.13E-04 | 40 |
| *POMC* | 2.66E+00 | 2.93E-04 | 5.07E-04 | 41 |
| *SHC2* | -6.59E-01 | 4.37E-04 | 7.39E-04 | 42 |
| *ATF6B* | 2.69E-01 | 7.61E-04 | 1.26E-03 | 43 |
| *MAP2K1* | 2.35E-01 | 7.96E-04 | 1.28E-03 | 44 |
| *KCNJ9* | 2.16E+00 | 1.79E-03 | 2.83E-03 | 45 |
| *NOS3* | -9.53E-01 | 4.81E-03 | 7.42E-03 | 46 |
| *ITPR3* | -2.51E-01 | 7.07E-03 | 1.07E-02 | 47 |
| *TFF1* | 4.22E+00 | 7.76E-03 | 1.15E-02 | 48 |
| *CREB1* | -1.48E-01 | 8.33E-03 | 1.21E-02 | 49 |
| *CREB5* | -6.10E-01 | 8.39E-03 | 1.19E-02 | 50 |
| *ADCY2* | 2.428339 | 1.01E-02 | 1.41E-02 | 51 |
| *NCOA2* | -0.28023 | 1.07E-02 | 1.47E-02 | 52 |
| *PIK3CD* | -0.47148 | 1.30E-02 | 1.74E-02 | 53 |
| *GRM1* | 2.86813 | 2.62E-02 | 3.44E-02 | 54 |
| *SHC4* | 1.087889 | 2.86E-02 | 3.69E-02 | 55 |
| *ESR1* | -0.59117 | 3.57E-02 | 4.53E-02 | 56 |
