# Supplementary material for: Transcriptome Based Estrogen Related Genes Biomarkers for Diagnosis and Prognosis in Non-small Cell Lung Cancer
Source: Front Genet. 2021 Apr 14;12:666396. doi: 10.3389/fgene.2021.666396 (PMC8081391; doi:10.3389/fgene.2021.666396)
Supplement: Supplementary file 5 [file Table_4.docx]

Table S4. Survival analysis of lung adenocarcinoma in TCGA database

| **Gene** | **HR** | ***p*** | **seq** | **FDR** |
| --- | --- | --- | --- | --- |
| *SHC1* | 1.436889 | 8.27E-07 | 1 | 4.14E-05 |
| *FKBP4* | 1.406697 | 4.10E-05 | 2 | 1.02E-03 |
| *NRAS* | 1.379923 | 1.77E-04 | 3 | 2.94E-03 |
| *PRKCD* | 0.667214 | 4.65E-04 | 4 | 5.81E-03 |
| *KRAS* | 1.1764 | 7.08E-04 | 5 | 7.08E-03 |
| *ADCY9* | 0.663067 | 2.14E-03 | 6 | 1.78E-02 |
| *PIK3CA* | 1.224258 | 3.37E-02 | 7 | 2.41E-01 |
| *ADCY8* | 1.14209 | 3.74E-02 | 8 | 2.34E-01 |
| *CREB5* | 1.167885 | 4.39E-02 | 9 | 2.44E-01 |
| *FKBP5* | 1.211986 | 6.80E-02 | 10 | 3.40E-01 |
| *CTSD* | 0.821073 | 9.05E-02 | 11 | 4.11E-01 |
| *MAP2K2* | 1.145463 | 1.02E-01 | 12 | 4.27E-01 |
| *ITPR3* | 0.858165 | 1.46E-01 | 13 | 5.60E-01 |
| *ADCY7* | 0.849987 | 1.77E-01 | 14 | 6.32E-01 |
| *HBEGF* | 1.121712 | 1.98E-01 | 15 | 6.60E-01 |
| *GRB2* | 1.136925 | 1.99E-01 | 16 | 6.22E-01 |
| *PIK3R2* | 0.881875 | 2.17E-01 | 17 | 6.39E-01 |
| *PIK3CB* | 1.112875 | 2.19E-01 | 18 | 6.08E-01 |
| *NCOA3* | 1.114051 | 2.25E-01 | 19 | 5.92E-01 |
| *NCOA1* | 0.897501 | 2.32E-01 | 20 | 5.79E-01 |
| *RARA* | 0.88616 | 2.33E-01 | 21 | 5.55E-01 |
| *AKT2* | 1.108736 | 2.38E-01 | 22 | 5.41E-01 |
| *SHC3* | 0.8893 | 2.83E-01 | 23 | 6.14E-01 |
| *ITPR1* | 0.894054 | 2.92E-01 | 24 | 6.08E-01 |
| *KCNJ6* | 0.874673 | 3.14E-01 | 25 | 6.27E-01 |
| *ESRRA* | 1.089871 | 3.17E-01 | 26 | 6.09E-01 |
| *CREB3* | 1.091419 | 3.29E-01 | 27 | 6.08E-01 |
| *SRC* | 0.898924 | 3.36E-01 | 28 | 6.01E-01 |
| *KRT1* | 0.80575 | 3.63E-01 | 29 | 6.25E-01 |
| *KCNJ5* | 0.873366 | 3.66E-01 | 30 | 6.10E-01 |
| *GNAS* | 0.932224 | 3.81E-01 | 31 | 6.14E-01 |
| *MAPK1* | 1.059107 | 4.26E-01 | 32 | 6.66E-01 |
| *MAPK3* | 0.92262 | 4.36E-01 | 33 | 6.60E-01 |
| *ATF6B* | 0.92406 | 4.86E-01 | 34 | 7.14E-01 |
| *ESR2* | 0.927902 | 5.12E-01 | 35 | 7.31E-01 |
| *PIK3R1* | 1.073273 | 5.22E-01 | 36 | 7.25E-01 |
| *MMP9* | 1.051651 | 5.78E-01 | 37 | 7.81E-01 |
| *CREB1* | 0.948391 | 5.87E-01 | 38 | 7.73E-01 |
| *OPRM1* | 0.95305 | 6.01E-01 | 39 | 7.71E-01 |
| *PIK3R3* | 0.958212 | 6.49E-01 | 40 | 8.11E-01 |
| *PGR* | 1.04531 | 6.55E-01 | 41 | 7.98E-01 |
| *FOS* | 0.961723 | 7.55E-01 | 42 | 8.99E-01 |
| *HRAS* | 1.027701 | 7.60E-01 | 43 | 8.84E-01 |
| *TFF1* | 0.971791 | 8.47E-01 | 44 | 9.62E-01 |
| *JUN* | 1.022469 | 8.56E-01 | 45 | 9.51E-01 |
| *ESR1* | 0.9756 | 8.80E-01 | 46 | 9.57E-01 |
| *NCOA2* | 1.013885 | 9.03E-01 | 47 | 9.60E-01 |
| *SHC2* | 0.989408 | 9.22E-01 | 48 | 9.60E-01 |
| *TGFA* | 1.010048 | 9.24E-01 | 49 | 9.43E-01 |
| *ADCY4* | 1.00274 | 9.80E-01 | 50 | 9.80E-01 |
