# Supplementary material for: Transcriptome Based Estrogen Related Genes Biomarkers for Diagnosis and Prognosis in Non-small Cell Lung Cancer
Source: Front Genet. 2021 Apr 14;12:666396. doi: 10.3389/fgene.2021.666396 (PMC8081391; doi:10.3389/fgene.2021.666396)
Supplement: Supplementary file 6 [file Table_5.docx]

Table S5. Survival analysis of lung squamous cell carcinoma in TCGA database

| **Gene** | **HR** | ***P*** | **seq** | **FDR** |
| --- | --- | --- | --- | --- |
| *MAPK3* | 1.277499 | 0.002004 | 1 | 0.11222 |
| *JUN* | 1.18704 | 0.007733 | 2 | 0.216514 |
| *CREB5* | 0.780485 | 0.011135 | 3 | 0.207863 |
| *ADCY7* | 1.239784 | 0.011232 | 4 | 0.157252 |
| *CTSD* | 1.180312 | 0.012089 | 5 | 0.135399 |
| *ESR1* | 1.186581 | 0.049876 | 6 | 0.465512 |
| *FOS* | 1.139018 | 0.080335 | 7 | 0.642684 |
| *ADCY3* | 0.857186 | 0.083308 | 8 | 0.58316 |
| *ITPR2* | 1.116716 | 0.113257 | 9 | 0.704712 |
| *RARA* | 1.139572 | 0.119669 | 10 | 0.670144 |
| *PRKCD* | 1.130256 | 0.133084 | 11 | 0.677518 |
| *RAF1* | 1.130029 | 0.133537 | 12 | 0.623171 |
| *NCOA2* | 1.128068 | 0.142178 | 13 | 0.612461 |
| *FKBP4* | 0.87302 | 0.157363 | 14 | 0.629453 |
| *FKBP5* | 1.136296 | 0.163049 | 15 | 0.608717 |
| *ADCY4* | 1.105997 | 0.18212 | 16 | 0.637419 |
| *AKT2* | 1.126119 | 0.18375 | 17 | 0.605294 |
| *PIK3CA* | 0.898493 | 0.195226 | 18 | 0.60737 |
| *MMP9* | 1.154645 | 0.208377 | 19 | 0.614165 |
| *ESRRA* | 1.11456 | 0.216204 | 20 | 0.605371 |
| *KCNJ5* | 1.080153 | 0.224621 | 21 | 0.598989 |
| *SRC* | 1.088347 | 0.284246 | 22 | 0.723536 |
| *TGFA* | 1.079396 | 0.301619 | 23 | 0.734377 |
| *GRM1* | 1.086131 | 0.321263 | 24 | 0.749613 |
| *NCOA3* | 1.080188 | 0.33447 | 25 | 0.749212 |
| *ATF6B* | 1.062677 | 0.378896 | 26 | 0.816083 |
| *PGR* | 1.073784 | 0.380773 | 27 | 0.789752 |
| *PIK3CD* | 1.062293 | 0.392203 | 28 | 0.784405 |
| *ITPR1* | 1.076331 | 0.394066 | 29 | 0.760954 |
| *TFF1* | 1.067076 | 0.399247 | 30 | 0.745261 |
| *EGFR* | 1.067237 | 0.426048 | 31 | 0.769635 |
| *KCNJ9* | 1.060625 | 0.433631 | 32 | 0.758855 |
| *CREB1* | 0.940517 | 0.435432 | 33 | 0.738914 |
| *KRAS* | 1.075811 | 0.459795 | 34 | 0.757309 |
| *NOS3* | 1.033072 | 0.52697 | 35 | 0.843151 |
| *PIK3R1* | 0.942726 | 0.528958 | 36 | 0.822824 |
| *PIK3R2* | 0.954276 | 0.53861 | 37 | 0.815194 |
| *GNAS* | 0.94918 | 0.540418 | 38 | 0.796406 |
| *NCOA1* | 1.050707 | 0.544035 | 39 | 0.781178 |
| *MAP2K2* | 1.046175 | 0.571375 | 40 | 0.799925 |
| *PIK3CB* | 1.044373 | 0.629765 | 41 | 0.860167 |
| *ADCY2* | 0.962274 | 0.650985 | 42 | 0.86798 |
| *ITPR3* | 1.03758 | 0.65627 | 43 | 0.854677 |
| *HBEGF* | 1.029764 | 0.727941 | 44 | 0.926471 |
| *SHC2* | 0.966487 | 0.737732 | 45 | 0.918066 |
| *SHC3* | 0.971704 | 0.74422 | 46 | 0.906007 |
| *POMC* | 0.971981 | 0.756425 | 47 | 0.901273 |
| *ADCY6* | 0.97619 | 0.777258 | 48 | 0.906801 |
| *HRAS* | 1.023324 | 0.779134 | 49 | 0.890439 |
| *PIK3R3* | 0.979297 | 0.800073 | 50 | 0.896082 |
| *SHC1* | 1.018638 | 0.828739 | 51 | 0.909988 |
| *NRAS* | 0.989073 | 0.885207 | 52 | 0.9533 |
| *SHC4* | 0.989547 | 0.891546 | 53 | 0.942011 |
| *MAP2K1* | 0.989283 | 0.913458 | 54 | 0.94729 |
| *GRB2* | 1.007146 | 0.927701 | 55 | 0.944568 |
| *ADCY9* | 0.994398 | 0.951275 | 56 | 0.951275 |
