# Supplementary material for: Transcriptome Based Estrogen Related Genes Biomarkers for Diagnosis and Prognosis in Non-small Cell Lung Cancer
Source: Front Genet. 2021 Apr 14;12:666396. doi: 10.3389/fgene.2021.666396 (PMC8081391; doi:10.3389/fgene.2021.666396)
Supplement: Supplementary file 7 [file Table_6.docx]

Table S6. Validation of survival analysis for lung adenocarcinoma in GSE68465

| Gene | HR | *P* |
| --- | --- | --- |
| *SHC1* | 0.957606 | 0.572 |
| *FKBP4* | 1.153404 | 0.035 |
| *NRAS* | 1.049727 | 0.239 |
| *PRKCD* | 0.99986 | 0.998 |
| *KRAS* | 1.233138 | 0.009 |
| *ADCY9* | 0.791963 | 0.012 |
